# The parasites of a successful invader: monogeneans of the Asian topmouth gudgeon *Pseudorasbora parva*, with description of a new species of *Gyrodactylus*[Fn FN1]

**DOI:** 10.1051/parasite/2023024

**Published:** 2023-06-17

**Authors:** Markéta Ondračková, Mária Seifertová, Maria Yu. Tkachenko, Lukáš Vetešník, Huanzhang Liu, Viktor Demchenko, Yuriy Kvach

**Affiliations:** 1 Institute of Vertebrate Biology of the Czech Academy of Sciences Květná 8 60365 Brno Czech Republic; 2 Department of Botany and Zoology, Faculty of Science, Masaryk University Kotlářská 2 61137 Brno Czech Republic; 3 The Key Laboratory of Aquatic Biodiversity and Conservation of the Chinese Academy of Science, Institute of Hydrobiology, Chinese Academy of Sciences Wuhan Hubei 430072 China; 4 Institute of Marine Biology, National Academy of Sciences of Ukraine 37 Pushkinska St. 65048 Odesa Ukraine

**Keywords:** *Bivaginogyrus*, Species invasion, Monogenea, New species, Parasite loss, Phylogeny

## Abstract

Monogenean parasites are often co-introduced with their fish hosts into novel areas. This study confirmed co-introduction of two dactylogyrids, *Dactylogyrus squameus* Gusev, 1955 and *Bivaginogyrus obscurus* (Gusev, 1955), and a newly described gyrodactylid species, *Gyrodactylus pseudorasborae* n. sp. into Europe along with their fish host, the invasive topmouth gudgeon *Pseudorasbora parva* (Temminck & Schlegel) from East Asia. All three species were observed in the lower Dnieper and middle Danube basin regions and had slightly larger haptoral hard parts than the same parasites in their native range. While dactylogyrids occurred sporadically, we recorded regular infection by *G. pseudorasborae* n. sp. at relatively high prevalence and abundance. This latter species was observed in both the native and non-native range of topmouth gudgeon, and resembles *Gyrodactylus parvae* You *et al.*, 2008 recently described from *P. parva* in China. Both species were distinguished based on genetic analysis of their ITS rDNA sequence (6.6% difference), and morphometric differences in the marginal hooks and male copulatory organ. Phylogenetic analysis of dactylogyrid monogeneans showed that *B. obscurus* clustered with *Dactylogyrus* species parasitising Gobionidae and Xenocyprididae, including *D. squameus*, supporting recent suggestions of a paraphyletic origin of the *Dactylogyrus* genus. In addition to co-introduced parasites, topmouth gudgeon was infected with a local generalist, *G. prostae* Ergens, 1964, increasing the number of monogeneans acquired in Europe to three species. Nevertheless, monogenean infections were generally lower in non-native host populations, potentially giving an advantage to invading topmouth gudgeon.

## Abstract (fr)

Les parasites monogènes sont souvent co-introduits avec leurs hôtes poissons dans de nouvelles zones. Cette étude a confirmé la co-introduction de deux Dactylogyridae, *Dactylogyrus squameus* Gusev, 1955 et *Bivaginogyrus obscurus* (Gusev, 1955), et d’une espèce de Gyrodactylidae nouvellement décrite, *Gyrodactylus pseudorasborae* n. sp. en Europe, en même temps que leur hôte poisson, le goujon envahissant *Pseudorasbora parva* (Temminck & Schlegel) qui vient d’Asie de l’Est. Les trois espèces ont été observées dans les régions du bas Dniepr et du bassin moyen du Danube et avaient des parties sclérifiées haptorales légèrement plus grandes que les mêmes parasites dans leur aire de répartition d’origine. Alors que les Dactylogyridae étaient rares, nous avons enregistré une infection régulière par *G. pseudorasborae* n. sp. à une prévalence et une abondance relativement élevées. Cette dernière espèce a été observée à la fois dans l’aire de répartition indigène et non indigène du goujon asiatique et ressemble à *Gyrodactylus parvae* You *et al.*, 2008 récemment décrit chez *P. parva* en Chine. Les deux espèces ont été distinguées sur la base de l’analyse génétique de leur séquence ITS de l’ADNr (différence de 6,6 %) et des différences morphométriques dans les crochets marginaux et l’organe copulateur mâle. L’analyse phylogénétique des monogènes Dactylogyridae a montré que *B. obscurus* se regroupait avec des espèces de *Dactylogyrus* parasitant les Gobionidae et les Xenocyprididae, y compris *D. squameus*, soutenant les suggestions récentes d’une origine paraphylétique du genre *Dactylogyrus*. En plus des parasites co-introduits, le goujon asiatique était infecté par un généraliste local, *G. prostae* Ergens, 1964, portant le nombre de monogènes acquis en Europe à trois espèces. Néanmoins, les infections par les monogènes étaient généralement plus faibles dans les populations d’hôtes non indigènes, donnant potentiellement un avantage à l’invasion du goujon asiatique.

## Introduction

The topmouth gudgeon *Pseudorasbora parva* (Temminck & Schlegel, 1846) is a freshwater cyprinid fish native to East Asia, including Japan, the Korean section of the Amur River basin, northern and central China and the southeastern part of Russia [3, 39]. The species was accidentally introduced into Europe in the 1960s with commercial fish imported from China and stocked in fishponds along the Romanian stretch of the Danube River [5]. Following early introductions into several European countries around the Black Sea (e.g., Romania, Hungary, Lithuania and Ukraine) in the early 1960s, the species rapidly became established in most waterbodies in Europe, parts of Eurasia and North Africa as a result of secondary introductions [82]. Recent genetic studies focused on identifying introduction pathways and colonisation history have indicated that topmouth gudgeon populations in Europe and Asia Minor result from two separate introduction events via two dispersal routes [31].

The majority of European topmouth gudgeon introduction events occurred accidentally along with stocking of Chinese carp (e.g., grass carp *Ctenopharyngodon idella* [Valenciennes in Cuvier & Valenciennes] and silver carp *Hypophthalmichthys molitrix* [Valenciennes, 1844]) for aquaculture and their further escape from fish farms [82], supplemented by occasional introductions either as ornamental fish [12] or as food for predatory fish in hatcheries [17]. Unlike intentional introductions, which can generally be controlled and managed through appropriate risk assessment, accidental introductions lack this regulatory procedure and, consequently, their risk can only be estimated retrospectively [26]. Such risks include disease transmission and co-introduction of new parasites and pathogens, which further increase the dangers of accidentally introduced non-native species for local fauna [86]. There are a range of examples of fish parasite co-introductions that have had detrimental effects on native fish species, most of which have shown as co-introduced parasites that are more virulent when successfully switching to native hosts (see [46]). One of the best documented examples is the introduction of the swim-bladder nematode *Anguillicola crassus* from Asia into Europe [37]. This nematode naturally infects Japanese eel (*Anguilla japonica*) at relatively low intensities, with no obvious adverse effects on fish physiology or condition [55]. After its successful introduction into Europe in the 1980s, however, the parasite switched to European eel *Anguilla anguilla* [37], reaching much greater infection intensities and causing severe pathological effects leading to mortality [22].

Reports on monogenean parasites co-introduced into Europe with topmouth gudgeon include two dactylogyrid species, *Dactylogyrus squameus* Gusev, 1955 and *Bivaginogyrus obscurus* (Gusev, 1955), and one gyrodactylid species, *Gyrodactylus gobioninum* Gusev, 1955. To date, *D. squameus* has been accidentally introduced from waterbodies in the Czech Republic [62, 78], Italy [23] and Ukraine [94], while single records of *B. obscurus* and *G. gobioninum* are known from Ukraine [94] and Bulgaria [48], respectively. In its native range, topmouth gudgeon is also parasitised by *Ancyrocephalus parvae* Achmerow, 1952 and *Gyrodactylus parvae* You *et al.*, 2008 [29, 58, 93]. Owing to their monoxenous life cycle, co-introduction of monogenean parasites is relatively common, especially in species translocated in higher numbers. There are many examples of monogeneans co-introduced into Europe, including for example *Dactylogyrus inexpectatus* and *D. dulkeiti* co-introduced with Prussian carp, *Carassius gibelio*; *D. lamellatus* with grass carp *Ctenopharyngodon idella*; *Pseudodactylogyrus bini* and *P. anguillae* with Japanese eel *Anguilla japonica* from Asia (summarised in [54]), and *Onchocleidus* spp., *Actinocleidus* spp., *Gyrodactylus centrarchi* and *Cleidodiscus robustus* with pumpkinseed sunfish *Lepomis gibbosus* [44, 65] and *Gyrodactylus nebulosus*, *G. melas*, *Ligictaluridus pricei* and *L. monticellii* with bullheads *Ameiurus nebulosus* and *A. melas* [64, 71, 90] from North-America. Most of these parasites are host specific and usually do not switch to other fish species. However, the two *Pseudodactylogyrus* species, for example, co-introduced with Japanese eel (*P. bini* and *P. anguillae*) were able to switch to European eel and after a relatively short period had increased their distribution throughout the European continent [84].

Though introduced species may serve as suitable hosts for local parasites, they are mostly generalists that take advantage of the “free niche” offered by the new hosts [68]. Monogeneans are among the most host-specific of parasites in general, and may be the most host-specific of all fish parasites [91]. Of these, *Dactylogyrus* species especially exhibit a high degree of host specificity [79], with potential host-switch usually only occurring between closely related cyprinid species (e.g. [80]). On the other hand, viviparous Gyrodactylidae are known to show a lower degree of host specificity [4]; thus, the introduction of a new competent host increases the chances of expanding the host spectrum for parasites with low host specificity. Interestingly, the topmouth gudgeon appears to be susceptible to a range of local generalist pathogens in its non-native range without showing clinical signs of pathology, suggesting that it is able to act as a healthy carrier for a number of pathogens [2]; nevertheless, just one report of infection by local monogeneans has been reported to date [48].

The main aim of this study was to identify monogenean parasites infecting topmouth gudgeon in the Czech Republic and at sites on the Dnieper River in Ukraine. We also compare parasite morphometric data obtained from non-native populations with parasites collected in the topmouth gudgeon’s native range, Chu-pej province in China, which is the presumed original source of fish introduction [82]. Using data from ribosomal DNA and morphometric analysis, we describe a new *Gyrodactylus* species introduced from Asia, which was found in both the Czech and Ukrainian regions. Finally, we provide insights into the phylogenetic relationships of parasites infecting topmouth gudgeon, with a special emphasis on their association with other East Asian species.

## Materials and methods

### Ethics

This research was undertaken in line with the ethical requirements of the Czech Republic and was approved by the appropriate ethics committee. The maintenance and care of fish, as well as the method of fish killing, complied with the legal requirements of the Czech Republic (§ 7 law No. 114/1992 on the Protection of Nature and Landscape and § 6, 7, 9 and 10 regulation No. 419/2012 on the Care, Breeding and Use of Experimental Animals).

### Fish sampling

Non-native topmouth gudgeon were collected by electrofishing in the Lower Dnieper irrigation system (Ukraine), the Kyjovka River and oxbows of the Morava and Dyje Rivers (Czech Republic). For comparative purposes, native fish were sampled from the Niushan and Bao’an Lakes (China; for locality details see Table 1). In total, 81 topmouth gudgeon were collected and transported alive in aerated cans to the nearby laboratory, where they were humanely dispatched and dissected for monogenean parasites within two days of capture, following Kvach *et al.* [42]. Prior to dissection, each fish was measured for standard length (SL) to the nearest 1 mm (Table 1).

**Table 1:** List of topmouth gudgeon *Pseudorasbora parva* sampling sites for monogenean assesment (2010 = native range, 2020–2022 = non-native range), showing coordinates, number of fish collected (n), fish host standard length range (SL, mm), and monogenean prevalence (%), intensity range (min-max) and mean abundance.

Locality/Country	Coordinates	*n*	SL	Sampling period	Monogenean species	Prevalence	Intensity range	Abundance
*Non-native range*								
Babice oxbow / Czech Republic	49.112634, 17.490357	20	28–71	September 2020	*Gyrodactylus pseudorasborae* n. sp.	15	2–3	0.4
Lower Dnieper irrigation system / Ukraine	46.272784, 32.734128	3	78–93	June 2021	*Gyrodactylus pseudorasborae* n. sp.	100	1–36	13.7
					*Bivaginogyrus obscurus*	33	4	1.3
					*Dactylogyrus squameus*	33	1	0.3
D3 oxbow / Czech Republic	48.684603, 16.916679	20	30–62	September 2021	*Gyrodactylus pseudorasborae* n. sp.	45	1–3	0.6
Melanbon pit / Czech Republic	48.676009, 16.923803	20	35–60	April 2022	*Gyrodactylus pseudorasborae* n. sp.	60	1–5	1.4
					*Gyrodactylus prostae*	20	1–4	0.4
					*Bivaginogyrus obscurus*	40	1–3	0.5
					*Dactylogyrus squameus*	5	1	0.1
River Kyjovka / Czech Republic	48.723237, 16.971280	11	39–75	May 2022	*Gyrodactylus pseudorasborae* n. sp.	36	5–17	3.2
					*Dactylogyrus squameus*	27	1–2	0.4
*Native range*								
Niushan Lake / China	30.347805, 114.522061	5	21–53	June 2010	*Gyrodactylus pseudorasborae* n. sp.	40	5–10	3.0
					*Bivaginogyrus obscurus*	100	1–14	7.2
					*Dactylogyrus squameus*	40	1	0.4
					*Ancyrocephalus parvae*	80	1–27	9.8
Bao’an Lake / China	30.237226, 114.729771	2	21–41	June 2010	*Gyrodactylus pseudorasborae* n. sp.	100	1–5	3.0

### Parasite collection and morphometric analysis

Gyrodactylid and dactylogyrid parasites collected from fins, gills, opercula and the body surface were mounted in glycerine-ammonium-picrate [47] as semi-permanent slides for morphological and morphometric analyses. Monogeneans dehydrated in ethanol and mounted in Canada Balsam are deposited in the Helminthological collection at the Institute of Parasitology, Academy of Sciences of the Czech Republic, České Budějovice (No: IPCAS-M-393, M-774, M-775). A subsample of monogeneans collected from the species’ non-native range was preserved in 96% ethanol for further molecular analysis.

Parasites were characterised according to the shape and size of the haptoral hard parts (anchors, connective bars, marginal hooks and/or copulatory organ) using a BX51 light microscope (Olympus Optical Co., Tokyo, Japan) equipped with phase-contrast and differential interference contrast. Drawings of haptoral hard components were made with the aid of a drawing attachment and phase-contrast optics. Measurements were obtained using the OLYMPUS cellSens Standard digital image analysis package (Olympus Optical Co., Hamburg, Germany). For gyrodactylids, 12 morphological characters of the anchors, ventral and dorsal bars, along with eight characters of the marginal hooks were measured according to Shinn *et al.* [76], supplemented by length and width of the whole body, pharynx and male copulatory organ (MCO). For dactylogyrids, 20 morphological characters of the anchors, ventral and dorsal bars, marginal hooks and copulatory organ were measured following Nitta and Nagasawa [57], supplemented by length and width of the whole body and pharynx (see Supplementary Tables S1, S2). For comparative morphometric analysis, 12 specimens of native *Gyrodactylus* sp., ten specimens of native *B. obscurus* and two specimens of *D. squameus* collected in China were subjected to the same morphometric procedure. Moreover, in addition to specimens obtained from topmouth gudgeon, we measured related *G. gobii* and *G. gobiensis* from *Romanogobio behlingi* and *Gobio gobio* collected in the Velička River as part of the study reported by Kvach *et al.* [43].

Prevalence (in %), intensity of infection (range, minimum–maximum) and mean abundance were calculated following Bush *et al.* [15]. A principal component analysis (PCA) based on standardised data was used to visualise the position of *Gyrodactylus* species in morphological space according to Dávidová *et al.* [21], applying measurements of 19 morphological characters. Owing to high inter-individual variation, the aperture distance was excluded from the PCA analysis. All statistical analyses were performed using Statistica v.14.1 (StatSoft Inc., USA; [85]).

### DNA extraction, amplification and sequencing

For parasites collected in Europe, the haptor of a previously ethanol-preserved parasite was excised and mounted in Hoyer’s medium for morphological confirmation of species, the remaining part of the body being placed in a 1.5 mL Eppendorf tube with 96% ethanol for genomic DNA extraction. Bisected parasite specimens preserved in 96% ethanol were dried using a centrifugal vacuum concentrator (Eppendorf, Hamburg, Germany). Genomic DNA was extracted separately from each parasite specimen using a DNeasy Blood & Tissue Kit (QIAGEN, Hilden, Germany), following the provided protocol for purification of total DNA from animal tissues.

For *Gyrodactylus* species, ten specimens of *Gyrodactylus* sp. from the Dnieper River (*n* = 5), D3 oxbow (*n* = 2) and Melanbon borrow pit (*n* = 3) were used for molecular analysis, along with two specimens of *G. prostae* from the Melanbon borrow pit (for information on sites, see Table 1). The region of the ribosomal DNA (rDNA) encompassing the 3′ end of 18S rDNA, ITS1, 5.8S rDNA, ITS2 and the 5′ end of 28S rDNA was amplified using the primers ITS1F (5′–GTTTCCGTAGGTGAACCT–3′) [74] and ITS2 (5′–TCCTCCGCTTAGTGATA–3′) [20]. The PCR reaction was performed at a final volume of 30 μL, including 5 μL of DNA extract (corresponding to 20 ng/μL), 1× PCR Buffer (Fermentas, Thermo Fisher Scientific, Waltham, MA, USA), 1.5 mM MgCl_2_, 200 μM dNTPs, 0.5 μM of each primer and 1.5 U Taq Polymerase (Fermentas). Cycling conditions were as follows: initial denaturation for 3 min at 96 °C, 35 cycles of 50 s at 95 °C, 50 s at 52 °C, 50 s at 72 °C and a final extension for 7 min at 72 °C.

For dactylogyrid species, one specimen of *D. squameus* from the Melanbon borrow pit, along with two specimens of *B. obscurus* from the Dnieper River and two specimens from the Melanbon borrow pit were used for molecular identification. The partial fragment of 28S rDNA was amplified using the primers C1 (5′–ACCCGCTGAATTTAAGCA–3′) and D2 (5′–TGGTCCGTGTTTCAAGAC–3′) [33], and a second fragment, spanning partial 18S rDNA, 5.8S rDNA and the entire ITS1 region (18S–ITS1), was amplified using the primers S1 (5′–ATTCCGATAACGAACGAGACT–3′) [83] and Lig5.8R (5′–GATACTCGAGCCGAGTGATCC–3′) [13]. The PCR reaction contained 5 μL of DNA extract, 1× PCR buffer (Fermentas, USA), 1.5 mM MgCl_2_, 0.5 μM (28S) or 0.8 μM (18S–ITS1) of each primer, 200 μM of each dNTP and 1 U of Taq polymerase (Fermentas, USA) at a final volume of 30 μL. The PCR was performed under the following conditions: initial denaturing step for 2 min at 94 °C; 39 cycles of denaturing for 60 s (18S–ITS1) or 20 s (28S) at 94 °C, annealing for 60 s at 50 °C (18S–ITS1) or for 30 s at 56 °C (28S), extending for 90 s at 72 °C and a final extending step for 10 min at 72 °C. All PCR products were electrophoresed on 1.5% agarose gels stained with GoodView (SBS Genetech, Bratislava, Slovakia) and then purified using ExoSAP-IT™ (Amplia, Bratislava, Slovakia), following the manufacturer’s protocol. The purified PCR products were sequenced directly in both directions using the same primers as in the amplification reaction. For ITS sequencing of *Gyrodactylus* species, an additional internal primer ITSR3A (5′–GAGCCGAGTGATCCACC–3′) [49], complementary to the sequence at the 5′ end of the 5.8S gene, was used. Sequencing was carried out using a BigDye^®^ Terminator v3.1 Cycle Sequencing Kit (Applied Biosystems, Thermo Fisher Scientific, Prague, Czech Republic) and an Applied Biosystems 3130 Genetic Analyser (Applied Biosystems, Thermo Fisher Scientific). The DNA sequences were assembled and edited using Sequencer software (Gene Codes Corp., Ann Arbor, MI, USA) and the newly generated monogenean sequences were deposited in GenBank (for accession numbers, see Tables 2 and 3.

**Table 2:** List of gyrodactylid taxa, host species, collection localities and GenBank accession numbers for ITS sequences used in the phylogenetical analysis. Newly generated sequences from the present study are highlighted in bold.

Species	Host species	Host family	Locality	Reference	GenBank accession number
**GYRODACTYLIDAE**					
*Gyrodactyloides bychowskii*	*Salmo salar*	Salmonidae	Scotland	[14]	AJ249348
*Gyrodactylus carassii*	*Carassius carassius*	Cyprinidae	Finland, Baltic Sea basin	[96]	AY278033
*Gyrodactylus elegans*	*Abramis brama*	Leuciscidae	Sweden, Baltic Sea basin	[96]	AY278034
*Gyrodactylus gobii*	*Gobio gobio*	Gobionidae	Czech Republic	[49]	AJ407873; AJ407922
*Gyrodactylus gobiensis*	*Gobio gobio*	Gobionidae	Poland, Baltic Sea basin	[96]	AY278041
*Gyrodactylus gracilihamatus*	*Gasterosteus aculeatus*	Gasterosteidae	Finland, Baltic Sea basin	[95]	AF484532
*Gyrodactylus gurleyi*	*Carassius auratus*	Cyprinidae	China	[45]	KC922453
*Gyrodactylus hrabei*	*Cottus poecilopus*	Cottidae	Slovakia	[92]	DQ288253
*Gyrodactylus jiroveci*	*Barbatula barbatula*	Nemacheilidae	Czech Republic	[70]	AM502860
*Gyrodactylus kobayashii*	*Carassius auratus*	Cyprinidae	China	[89]	KJ524572
*Gyrodactylus laevis*	*Phoxinus phoxinus*	Leuciscidae	Finland, White Sea basin	[96]	AY278036
*Gyrodactylus leucisci*	*Leuciscus leuciscus*	Leuciscidae	Finland, Baltic Sea basin	[95]	AF484536
*Gyrodactylus longipes*	*Sparus aurata*	Sparidae	Italy	[66]	GQ150536
*Gyrodactylus longiradix*	*Gymnocephalus cernua*	Percidae	Finland, Baltic Sea basin	[95]	AF484538
*Gyrodactylus luciopercae*	*Perca fluviatilis*	Percidae	Finland, Baltic Sea basin	[95]	AF484541
*Gyrodactylus markakulensis*	*Gobio gobio*	Gobionidae	Czech Republic	[49]	AJ407886; AJ407932
*Gyrodactylus ouluensis*	*Rutilus rutilus*	Leuciscidae	Finland, Baltic Sea basin	[41]	AF484546
*Gyrodactylus parvae*	*Pseudorasbora parva*	Gobionidae	China-Qinling Mountains	[93]	EF450249
*Gyrodactylus phoxini*	*Phoxinus phoxinus*	Leuciscidae	Finland, Baltic Sea basin	[96]	AY278037
***Gyrodactylus prostae***	*Pseudorasbora parva*	Gobionidae	Czech Republic	present study	**OQ598691**
*Gyrodactylus prostae*	*Rutilus rutilus*	Leuciscidae	Finland, Baltic Sea basin	[96]	AY278038
***Gyrodactylus pseudorasborae* n. sp.**	*Pseudorasbora parva*	Gobionidae	Czech Republic, Ukraine	present study	**OQ598690**
*Gyrodactylus pungitii*	*Pungitius pungitius*	Gasterosteidae	Belgium, North Sea basin	[97]	AF328869
*Gyrodactylus robustus*	*Platichthys flesus*	Pleuronectidae	Poland, Baltic Sea basin	[96]	AY278040
*Gyrodactylus salaris*	*Oncorhynchus mykiss*	Salmonidae	Finland	[97]	AF328871
*Gyrodactylus* sp.	*Pseudorasbora parva*	Gobionidae	China: Guilin	Chen *et al.*, 2022 (unpublished)	OP577877
*Gyrodactylus sprostonae*	*Carassius auratus*	Cyprinidae	China: River Irtysh	Zhang 2014 (unpublished)	KP295469

**Table 3:** List of dactylogyrid taxa, host species, collection localities and GenBank accession numbers for the 28S rDNA sequences used in the phylogenetical analysis. Newly generated sequences from the present study are highlighted in bold.

Species	Host species	Host family	Locality	Reference	GenBank accession number
**DACTYLOGYRIDAE**					
***Bivaginogyrus obscurus***	*Pseudorasbora parva*	Gobionidae	Ukraine, Czech Republic		**OQ598694**
*Dactylogyrus anchoratus*	*Carassius gibelio*	Cyprinidae	Croatia	[8]	KY863555
*Dactylogyrus bicornis*	*Rhodeus meridionalis*	Acheilognathidae	Greece-Pinios	[81]	KY629345
*Dactylogyrus bicorniculus*	*Rhodeus atremius*	Acheilognathidae	Japan-Saga	[59]	LC093099
*Dactylogyrus carassobarbi*	*Carasobarbus luteus*	Cyprinidae	Iraq	[11]	MZ031060
*Dactylogyrus clavaeformis*	*Hemiculter leucisculus*	Xenocyprididae	China	Chang *et al.*, 2022 (unpublished)	OP320920
*Dactylogyrus cornu*	*Vimba vimba*	Leuciscidae	Czech Republic	[81]	KY629371
*Dactylogyrus cryptomeres*	*Gobio gobio*	Gobioninae	Czech Republic	[79]	AJ969947
*Dactylogyrus extensus*	*Cyprinus carpio*	Cyprinidae	Czech Republic	[79]	AJ969944
*Dactylogyrus fallax*	*Vimba vimba*	Leuciscidae	Czech Republic	[81]	KY629370
*Dactylogyrus folkmanovae*	*Squalius cephalus*	Leuciscidae	Bosnia and Herzegovina	[9]	MG793028
*Dactylogyrus hypophthalmichthys*	*Hypophthalmichthys molitrix*	Xenocyprididae	China	Wu *et al.*, 2006 (unpublished)	EF100532
*Dactylogyrus inexpectatus*	*Carassius auratus*	Cyprinidae	Czech Republic	[79]	AJ969945
*Dactylogyrus kikuchii*	*Lateolabrax japonicus*	Lateolabracidae	China	Ding and Liao, 2004 (unpublished)	AY548929
*Dactylogyrus lamellatus*	*Ctenopharyngodon idella*	Xenocyprididae	Czech Republic	[79]	AJ969948
*Dactylogyrus latituba*	*Hemiculter leucisculus*	Xenocyprididae	China	Guo*et al.*, 2019 (unpublished)	MK353163
*Dactylogyrus lenkoranoides*	*Luciobarbus graellsii*	Cyprinidae	Spain	[10]	MN338211
*Dactylogyrus parabramis*	*Megalobrama terminalis*	Xenocyprididae	China	Wu *et al.*, 2006 (unpublished)	EF100534
*Dactylogyrus pseudogobii*	*Abbottina rivularis*	Gobionidae	China	Zhang and Fan, 2020 (unpublished)	MT997189
*Dactylogyrus primarius*	*Opsariichthys bidens*	Xenocyprididae	China	Chang *et al.*, 2022 (unpublished)	OP320903
*Dactylogyrus mascomai*	*Luciobarbus graellsii*	Cyprinidae	Spain	[10]	MN338215
*Dactylogyrus quanfami*	*Cirrhinus molitorella*	Cyprinidae	China	Wu *et al.*, 2006 (unpublished)	EF100536
*Dactylogyrus sphyrna*	*Vimba vimba*	Leuciscidae	Czech Republic	[9]	MG793066
*Dactylogyrus squameus*	unknown; *Pseudorasbora parva*	Gobionidae	China; Czech Republic	Fan *et al.*, 2016 (unpublished); [15]	KX812459
***Dactylogyrus squameus***	*Pseudorasbora parva*	Gobionidae	Czech Republic		**OQ598695**
*Dactylogyrus suchengtaii*	*Hypophthalmichthys molitrix*	Xenocyprididae	Iran: Guilan	Daghigh *et al.*, 2018 (unpublished)	MG825765
*Dactylogyrus vastator*	*Carassius gibelio*	Cyprinidae	Croatia	[11]	MW443031
*Ancyrocephalus percae*	*Perca fluviatilis*	Percidae	Germany	[6]	KF499080
*Gobioecetes biwaensis*	*Rhinogobius* sp.	Gobiidae	Japan-Shiga	[60]	LC494515
*Gobioecetes longibasis*	*Rhinogobius similis*	Gobiidae	Japan-Okinawa	[60]	LC494516
*Pseudodactylogyrus anguillae*	*Anguilla anguilla*	Anguillidae	Austria	[79]	AJ969950
*Pseudodactylogyrus bini*	*Anguilla anguilla*	Anguillidae	Austria	[79]	AJ969949

### Phylogenetic analysis

Both *Gyrodactylus* and *Dactylogyrus* phylogenies were analysed using Maximum Likelihood (ML) and Bayesian inference (BI) methods. Additional monogenean sequences for phylogenetic analysis were retrieved from GenBank (for accession numbers, see Tables 2 and 3). The *Gyrodactylus* dataset for the ITS rDNA sequences included three species parasitising topmouth gudgeon (*Gyrodactylus pseudorasborae* n. sp., *G. parvae* and *G. prostae*) and 19 selected *Gyrodactylus* species mainly collected from fish hosts in the Eurasian regions and from phylogenetically related hosts (Gobioninae) comprising three main lineages previously observed in *Gyrodactylus* phylogeny [25, 50, 96]. The species *Gyrodactyloides bychowskii* Albova, 1948 was used as outgroup (Table 2).

To determine the phylogenetic position of *B. obscurus* within Dactylogyridae, a 28S rDNA dataset, including the newly generated sequences for dactylogyrids parasitising topmouth gudgeon and those available from GenBank representing the four main phylogenetic lineages of *Dactylogyrus* according to Šimková *et al.* [81], was created and analysed. The five dactylogyridean species representing three genera (*Ancyrocephalus*, *Gobioecetes* and *Pseudodactylogyrus*) were selected as an outgroup (Table 3).

All alignments were performed with MAFFT v.7 [36], using the “G-INS-I” strategy and optimised manually in BioEdit [30]. ModelFinder [35] was employed to infer the optimal evolutionary model for each genetic segment using the Bayesian information criterion. The following optimal evolutionary models were selected: TVM + F + G for ITS1, TIM2e + G for 5.8S and TVM + F + G for ITS2 in *Gyrodactylus* phylogeny; GTR + F + I + G for 28S rDNA in Dactylogyridae phylogenetic reconstruction. ML analysis was conducted using the program IQ-TREE [56], as implemented in W-IQ-TREE [88], with nodal support assessed through 1,000 ultrafast bootstrap (“UFBoot”) replicates [52]. BI analysis was performed using MrBayes v. 3.2.1 [75], using four simultaneous chains (one cold and three heated) of the Markov Chain Monte Carlo (MCMC) algorithm run twice for 1,000,000 generations. Tree topologies were sampled every 100 generations, whereby the first 25% of trees from each run were discarded as burn-in. Convergence was indicated by an average standard deviation of split frequencies per parallel run of < 0.01, and subsequently checked using Tracer v.1.7.1. [73]). The resulting trees were visualised and edited in FigTree v.1.4.3. [72]. Finally, the genetic divergence between morphologically similar species was calculated using uncorrected p-distances for each genetic segment in MEGA X [40].

## Results

### 1 Gyrodactylidae

Two species, *G. prostae* Ergens, 1964 and *G. pseudorasborae* n. sp. were recorded within the topmouth gudgeon non-native range. *Gyrodactylus prostae* was found on a fish’s gills at a single locality, the Melanbon borrow pit (Dyje River floodplain) in April 2022, with prevalence of 20%, abundance 0.4 and intensity of infection ranging between 1 and 4 (Table 1). The size of the marginal hooks corresponded to *G. prostae* sensu Galli *et al.* [24], while the size of the anchors was smaller and corresponded to the related *G. laevis* (Table 4). Identical sequences of the 886 bp ITS rDNA fragment (submitted to the GenBank database under accession number OQ598691) were obtained from two specimens. A BLASTn search (February 2023) showed maximum similarity to *G. prostae* from cyprinids in the Czech Republic (100%; AJ567673; [50]) and Finland (99.89%; AY278038; [96]).

**Table 4:** Morphometric parameters (mean, range; μm) of *Gyrodactylus prostae* collected from topmouth gudgeon *Pseudorasbora parva* in the Melanbon borrow pit, Czech Republic, compared with published data.

	*Gyrodactylus prostae ex Pseudorasbora parva* Melanbon, CR; *n* = 6	*Gyrodactylus prostae* Galli *et al.* [24]	*Gyrodactylus laevis* Galli *et al.* [24]
Body length	507 (413–557)	300–600	350
Body width	90 (75–113)		
Haptor length	98 (84–111)		
Haptor width	111 (75–160)		
Pharynx length	33 (29–36)		
Pharynx width	35 (35–35)		
Penis length	13 (*n* = 1)		
Penis width	13		
Spines	1/6 + 3		
*Anchor*			
Total length	39 (37–41)	44–60	33–43
Shaft	28 (25–30)	34–44	27–34
Root	13 (10–15)	16–22	10–16
Point	18 (16–20)	20–30	15–18
Aperture distance	18 (13–24)		
*Ventral bar*			
Total width	15 (14–18)	15–19	9–15
Total length			
Median length	4.7 (3.7–5.6)	4–7	4–6
Membrane length	11 (8.0–13)	12–16	9–16
Ventral bar process to mid length	1.5 (1.0–2.5)		
*Dorsal bar*			
Total length	10 (9.1–12)	9–11	7–9
Width	1.7 (1.4–1.9)	2–3	1
*Marginal hook*			
Total length	24 (24–25)	24–30	15–20
Shaft	18 (17–19)		
Sickle length	6.8 (6.3–7.3)	7–8	5–6
Distal width	2.7 (2.5–3.0)		
Proximal width	4.1 (3.7–4.2)		
Aperture distance	5.2 (4.7–5.7)		
Toe width	1.7 (1.5–1.9)		
Filament length	14 (13–15)		

**Figure 1:**
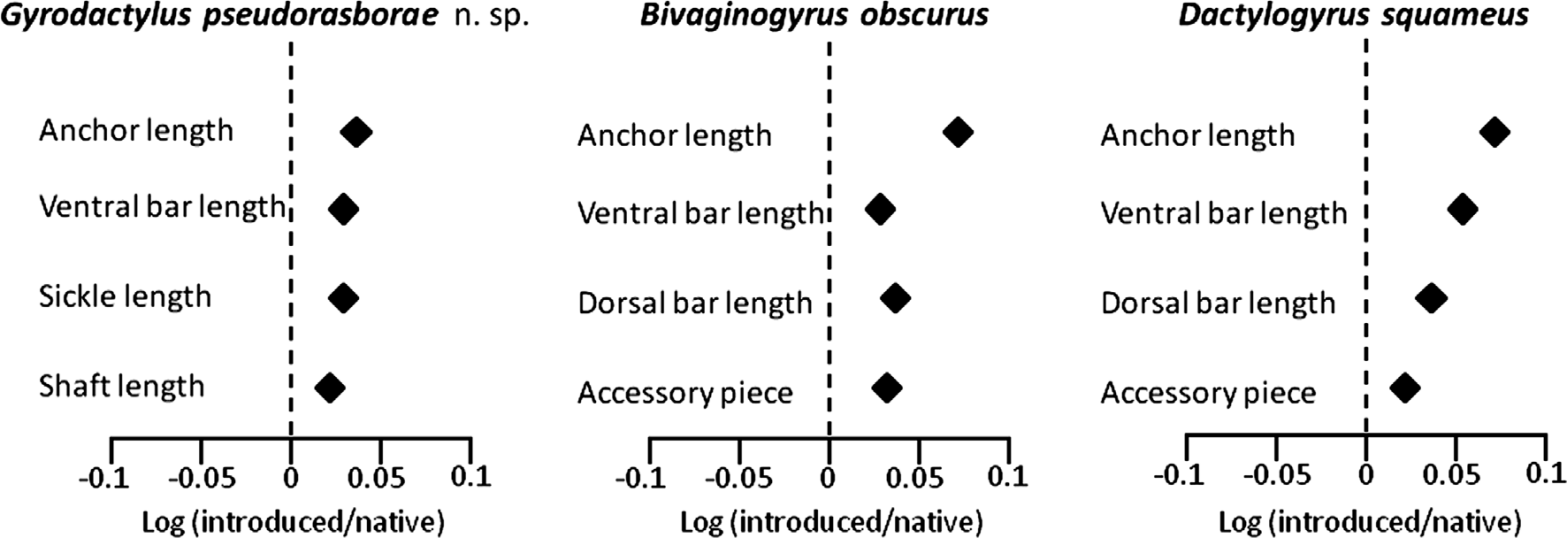
Response ratios depicting the mean length of selected parameters for monogenean hard parts (length of anchor, ventral bar, sickle and shaft for gyrodactylids; length of anchor, ventral and dorsal bars and accessory piece of copulatory organ for dactylogyrids). The *x*-axis is the log of the ratio of measurements in introduced vs. native ranges. Positive numbers indicate enhanced performance in the introduced range.

**Figure 2:**
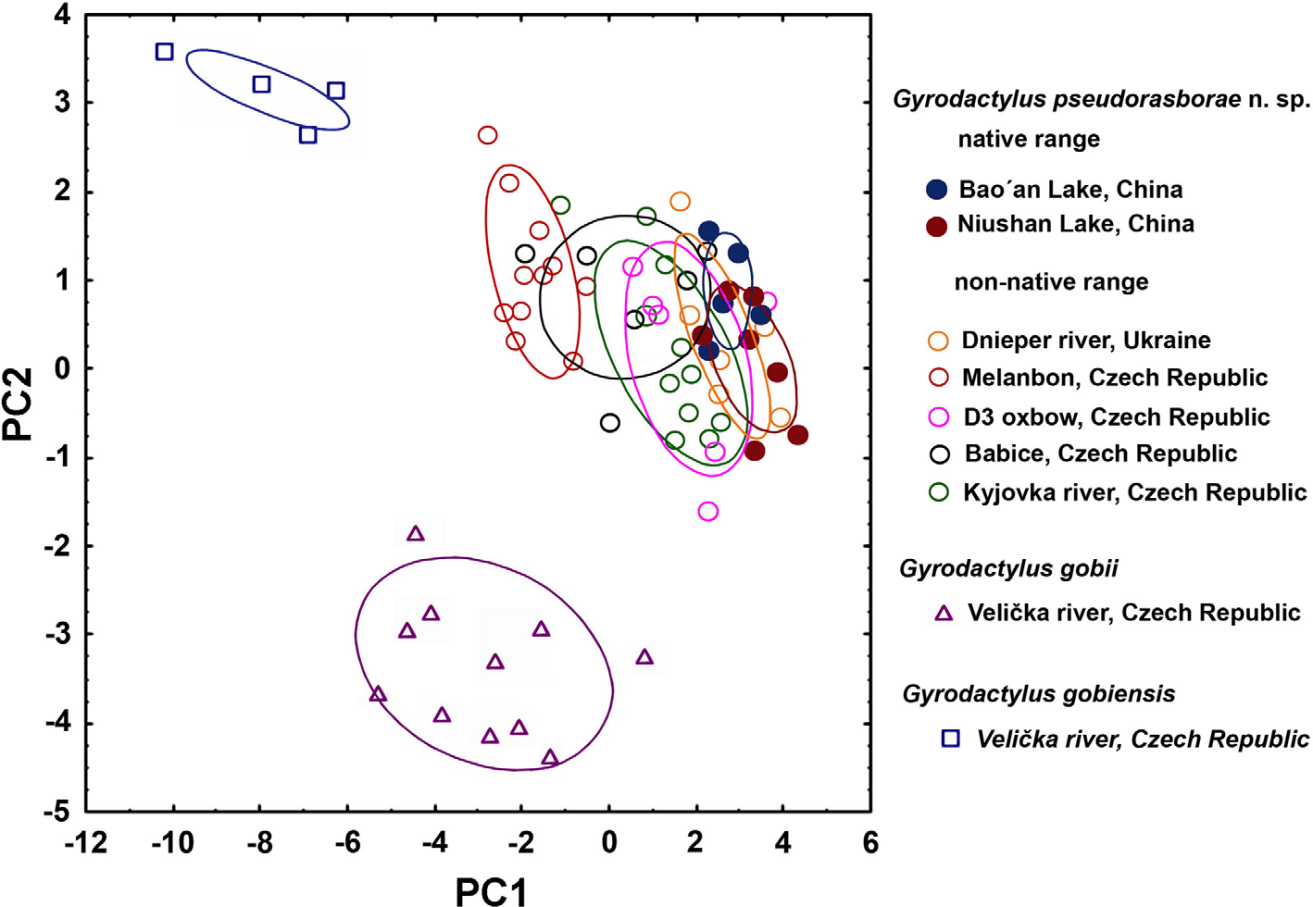
Plot of a principal component analysis based on 19 metric characters for the haptoral hard parts. The plot shows the position of *Gyrodactylus pseudorasborae* n. sp. obtained from *Pseudorasbora parva* in its native Asian (China; full circles) and non-native European (Czech Republic, Ukraine; empty circles) ranges, along with related *G. gobii* (empty triangles) obtained from *Romanogobio vladykovi*, and *G. gobiensis* (empty squares) obtained from *Gobio gobio* in the Czech Republic, in morphological space. Ellipses covering 95% range.

A morphologically similar *Gyrodactylus* species that did not correspond to any other *Gyrodactylus* species known from topmouth gudgeon or related fish hosts was found at all European sampling localities (non-native range) and China (native range). Specimens from localities in both native and non-native ranges varied in size and largely overlapped in the measurements of haptoral hard parts (Supplementary Table S1). However, the mean size of haptoral hard parts was larger in introduced worms (Fig. 1). As an exception, all gyrodactylids collected from the Melanbon borrow pit exhibited larger sizes in most features than those from other sites (Supplementary Table S1), and were separated from other localities (i.e., Dnieper and Kyjovka Rivers, D3 oxbow and the Bao’an and Niushan Lakes) on the PCA plot along the PC1 axis (Fig. 2). All sequences of the ITS rDNA fragment obtained from 10 specimens collected from the Dnieper River, the D3 oxbow and the Melanbon borrow pit were identical. A BLASTn search (February 2023) revealed highest similarity with the ITS rDNA sequences of *Gyrodactylus* sp. (99.57%; OP577877; Chen *et al.* unpublished) and *Gyrodactylus parvae* (93.43%; EF450249; [93]), obtained from native topmouth gudgeon sampled in the Qinling mountains of central China. The parasite is therefore described as a new species.

### Description of the new species

Family Gyrodactylidae Cobbold, 1864

Genus *Gyrodactylus* von Nordmann, 1832

#### *Gyrodactylus pseudorasborae* n. sp. Ondračková, Seifertová & Tkachenko (Fig. 3)

urn:lsid:zoobank.org:act:78975C09-695F-4C2F-ACA2-51EB6D8ACFC8

**Figure 3:**
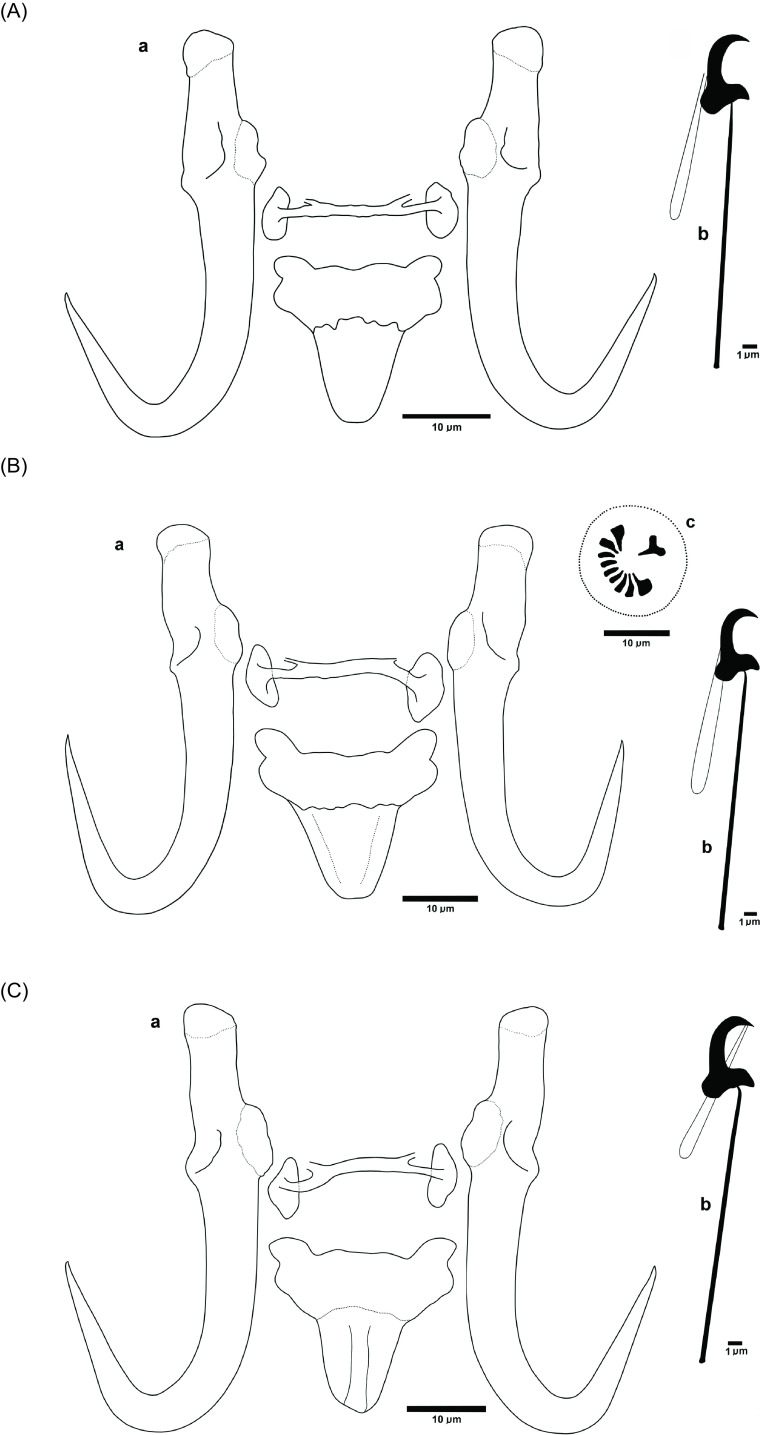
Line drawings of *Gyrodactylus pseudorasborae* n. sp. ex *Pseudorasbora parva*. A = Holotype (China), B = Paratype (Ukraine), C = Paratype (Czech Republic). a = opisthaptoral central hook complex; b = marginal hook; c = male copulatory organ. Scale bar = 10 μm.

*Type host*: *Pseudorasbora parva* (Temminck & Schlegel, 1846), Cypriniformes

*Site on the host*: fins, gills, opercula, body surface

*Type locality*: Bao’an Lake, Hubei Province (30.237226, 114.729771), China

*Other localities*: Dnieper River basin (46.272784, 32.734128), Ukraine; Babice oxbow, Morava basin (49.122634, 17.490357); D3 oxbow (48.684603, 16.916679), Melanbon borrow pit, Dyje basin (48.676099, 16.923803); Kyjovka River (48.723237, 16.971280); Danube basin, Czech Republic; Niushan Lake, Hubei Province (30.347805, 114.522061) China.

*Type specimens*: Holotype and three paratypes mounted in Canada Balsam, and three hologenophores, deposited in the Helminthological collection at the Institute of Parasitology, Academy of Sciences of the Czech Republic, České Budějovice (IPCAS M-775). Two paratypes deposited in the Natural History Museum in Vienna, Austria (NHMW-ZOO-EV-M-5883, NHMW-ZOO-EV-M-5884), and two parasites deposited in Hasselt University in Diepenbeek, Belgium (HU XIX.2.21, HU XIX.2.22).

*Etymology*: The specific epithet has a root from the fish host genus name, *Pseudorasbora*.

*Note:* The authors of the new taxa are different from the authors of this paper: Article 50.1 and Recommendation 50A of the International Code of Zoological Nomenclature [34].

*Material examined*: 58 flattened specimens (morphology; 5 specimens from type locality), 10 ethanol preserved specimens (DNA analysis, Europe).

##### Morphological description (Fig. 3, Table 5)

General morphology based on 58 specimens (i.e., *n* = 58, unless otherwise stated). Measurements from different sampling sites are shown in Supplementary Table S1. Body small to medium, elongate, with length 549 (299–703) and width 116 (72–187) at midbody (*n* = 52). Haptor circular to oval, 116 (71–181) long, 99 (60–142) wide, pharynx 34 (20–50) long and 33 (21–47) wide (*n* = 52). MCO circular, located laterally to pharynx, 15 (10–18) long and 15 (13–20) wide, observed in 12 specimens at five localities. MCO armed with one principal spine, two medium spines and 6–7 smaller spines in a single row (Fig. 3B–c). Anchors robust, total length 51.9 (46.2–59.0), shaft length 34.6 (29.2–39.6), root length 16.4 (11.9–24.2), point length 23.6 (18.5–27.9), and aperture length 17.8 (13.0–24.5; *n* = 57). Dorsal bar 1.9 (1.2–2.6) long, 19.9 (17.1–25.4) wide, with protrusions at the top on either side of the bar, about one-quarter from the attachment to the anchor. Ventral bar (*n* = 55) median length 6.8 (5.2–8.3), total length 21.9 (18.1–27.5), width 23.0 (19.1–27.6), anterolateral processes of ventral bar 2.7 (1.5–4.0) long. Membrane 12.1 (9.4–16.4) long, subrectangular, tongue tapering. Marginal hook total length 25.0 (22.8–27.4), shaft length 20.0 (18.2–22.3). Marginal hook sickles 5.2 (4.5–5.9) long, 2.6 (2.0–3.1) wide distally, 3.4 (2.7–3.9) wide proximally, aperture 4.1 (3.5–4.8) long; sickle toe 1.2 (0.9–1.4) wide, filament 11.0 (9.6–12.7) long.

**Table 5:** Comparison of morphometric parameters (mean, range; μm) for *Gyrodactylus pseudorasborae* n. sp. collected in various localities in Europe and China with other closely related monogenean species.

	*Gyrodactylus pseudorasborae* n. sp. ex *P. parva*	*Gyrodactylus parvae* ex *P. parva* You *et al.* [93]	*Gyrodactylus gobioninum*; Galli *et al.* [24]	*Gyrodactylus gobioninum*; Margaritov and Kiritsis [48]	*Gyrodactylus gobii* ex *Romanogobio vladykovi* River Velička, CR	*Gyrodactylus gobiensis* ex *Gobio gobio* River Velička, CR
	*n* = 58	*n* = 10		*n* = 41	*n* = 12	*n* = 3
Body length	549 (299–703)	490–802	300		751 (644–828)	660 (581–778)
Body width	116 (72–187)	44–99			121 (86–148)	123 (90–143)
Haptor length	99 (60–142)	51–92			102 (91–121)	106 (95–127)
Haptor width	116 (71–181)	30–121			135 (112–150)	122 (107–140)
Pharynx length	34 (21–47)	25–39			51 (42–63)	33 (28–38)
Pharynx width	33 (20–50)				51 (42–63)	32 (28–37)
Penis length	15 (10–18)	14.7			18 (16–20)	16 (14–19)
Penis width	15 (13–20)				18 (15–21)	15 (13–18)
Spines	1/5–7	1 + 2/2 + 5			1/7–8	
*Anchor*						
Total length	52 (46–59)	47–54	51	52–60	52 (50–57)	66 (61–68)
Shaft	35 (29–40)	35–41	37	38–45	35 (34–37)	43 (40–47)
Root	16 (12–24)	14–20	16	15–20	16 (15–18)	20 (18–22)
Point	24 (19–28)	22–27	26	23–28	27 (25–28)	32 (31–33)
Aperture distance	18 (13–25)				17 (15–22)	21 19–23)
*Ventral bar*						
Total width	23 (19–28)	19–26	17	23–28	24 (21–26)	28 (27–29)
Total length	22 (18–27)				23 (19–25)	28 (27–29)
Median length	6.8 (5.2–8.3)	3.4–7.4	6	6–8	6.0 (4.7–7.3)	7.7 (7.6–7.9)
Membrane length	12 (9.4–16)	8.8–14.4	12	13–18	13 (11–15.5)	17 (15–18)
Ventral bar process to mid length	2.7 (1.5–4.0)	1.5–2.8			3.7 (2.4–5.1)	3.6 (3.2–4.3)
*Dorsal bar*						
Total length	20 (17–25)	16–19	17	18–25	19 (17–21)	25 (23–27)
Width	1.9 (1.2–2.6)	1.4–2.6	2	2–3	2.1 (1.7–2.7)	1.9 (1.6–2.2)
*Marginal hook*						
Total length	25 (23–27)	30–31.7	24	24–27	27 (26–28)	29 (28–32)
Shaft	20 (18–22)	25–26.9			21 (20–22)	23 (22–25)
Sickle length	5.2 (4.5–5.9)	4.9–5.7	5	5–6	6.4 (5.9–7.1)	6.3 (6.0–6.6)
Distal width	2.6 (2.0–3.1)	2.7–3.9			4.0 (3.2–4.9)	3.6 (3.3–3.7)
Proximal width	3.4 (2.7–3.9)	3.1–4.0			4.5 (4.1–5.1)	3.8 (3.5–4.2)
Aperture distance	4.1 (3.5–4.8)				4.7 (4.1–5.1)	5.1 (4.7–5.6)
Toe width	1.2 (0.9–1.4)				1.7 (1.4–2.1)	1.2 (1.0–1.5)
Filament length	11 (9.6–13)	10.2–13.5			12 (11–13.5)	11 (10–12)

##### Representative DNA sequence

The 1233 bp sequence comprising the partial 18S rDNA (15 bp) and 28S rDNA (9 bp), and complete ITS1 (625 bp), 5.8S rDNA (157 bp) and ITS2 (427 bp) sequences, have been deposited in the GenBank database under accession number OQ598690.

#### Remarks

**Figure 4:**
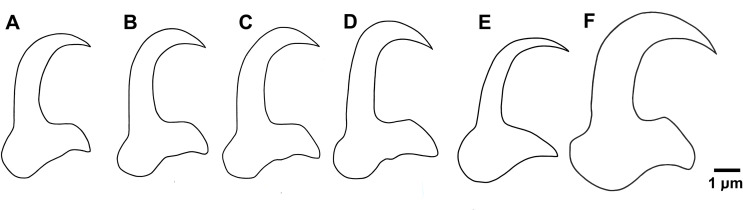
A–C: Line drawings of the marginal hook sickle of *Gyrodactylus pseudorasborae* n. sp. ex *Pseudorasbora parva* from China (A), Ukraine (B) and the Czech Republic (C); D: *G. parvae* ex *Pseudorasbora parva*, China, redrawn from You *et al.* (2008); E: *G. gobioninum*, redrawn from Galli *et al.* 2010, and F: *G. gobii* ex *Romanogobio vladykovi*, from the Czech Republic.

The morphology and size of the haptoral hard parts of *G. pseudorasborae* n. sp. are closely related to those of *G. parvae*, recently described from topmouth gudgeon in China [93], and to *G. gobiensis* and *G. gobii* parasitising various gudgeon species in Eurasia [24, 77]. The size of the anchors is comparable with those of *G. parvae* and *G. gobii*, though it is smaller than those of *G. gobiensis* ([24]; Table S1). *Gyrodactylus pseudorasborae* n. sp. differs from the other *Gyrodactylus* species in features related to the marginal hook, dorsal bar and MCO. Based on morphology and metrics of the marginal hooks, the new species differs (1) from *G. parvae* by the length of the marginal hook shaft and width of the marginal sickle toe, being smaller in *G. pseudorasborae* n. sp.; (2) from *G. gobii* by the sickle length, distal and proximal sickle width and toe width, which are larger in *G. gobii*; and (3) from *G. gobioninum* by the shape of the sickle, which is more straight and robust in *G. pseudorasborae* n. sp. (Fig. 4). The morphology of the dorsal bar, i.e., the presence of protrusions at the top on either side of the bar, resembles that of *G. gobioninum*, but differentiates the new species from *G. gobii*. Unfortunately, a drawing of the dorsal bar was not provided in the description of *G. parvae* and, moreover, the bar was not in focus in the microphotograph published in [93], making morphology of the dorsal bar unclear in *G. parvae*. Finally, *G. pseudorasborae* n. sp. differs from the other species in the number of spines on the MCO. While it consists of one large, two medium and 6–7 small spines in *G. pseudorasborae* n. sp., the MCO in *G. parvae* and *G. gobii* consists of one large, two medium and five small spines, and one large, two medium and three small spines in *G. gobioninum* [24, 93]. The ITS sequence data obtained, and their phylogenetic associations, support the designation of a new species, discriminating *G. pseudorasborae* n. sp. from the morphometrically similar species *G. parvae* and *G. gobii*.

#### *Phylogenetic positioning of* Gyrodactylus pseudorasborae *n. sp.*

**Figure 5:**
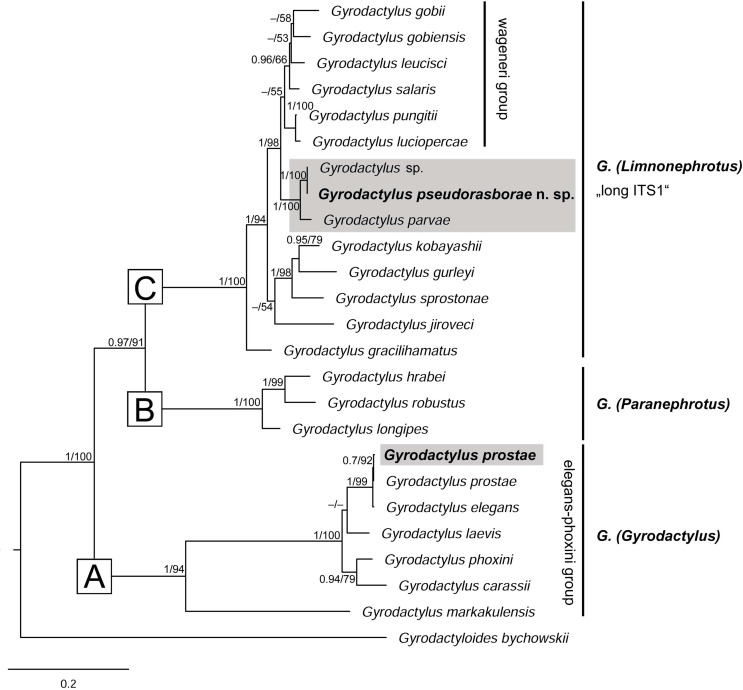
The phylogenetic relationships of *Gyrodactylus pseudorasborae* n. sp. and *G. parvae* parasitising *Pseudorasbora parva* within related Eurasian *Gyrodactylus* species. The phylogenetic tree is inferred from a Maximum Likelihood (ML) analysis of the 834 bp alignment of ITS1-5.8S rDNA-ITS2 sequences. Numbers along branches represent Bayesian posterior probability/ML bootstrap support (only values > 0.70 for BI and > 50% for ML shown). Newly obtained sequences from the present study are in bold. Sequences of *Gyrodactylus* species parasitising *Pseudorasbora parva* are highlighted in grey. The branch length scale bar indicates the number of substitutions per site.

ML and BI analyses of ITS1-5.8S rDNA-ITS2 sequences yielded almost identical trees, with the topology roughly corresponding to the tree structure in Gilmore *et al.* [25], Zietara *et al.* [96] and Matějusová *et al.* [50]. *Gyrodactylus* species were found to form three major clades (Fig. 5), the new sequences of both *Gyrodactylus* species obtained in the present study being nested within two of the three recovered clades. Sequence data support the position of *G. pseudorasborae* n. sp. within the “long” ITS1 clade (sensu Cable *et al.* [16]) consisting of Eurasian species belonging to the subgenus *Limnonephrotus*. *Gyrodactylus pseudorasborae* n. sp., *Gyrodactylus* sp. and *G. parvae*, all parasites of topmouth gudgeon, were shown to be sister taxa and formed a well-supported clade in a basal position to the “wageneri” group, consisting of *G. gobii*, *G. gobiensis*, *G. leucisci*, *G. longiradix*, *G. luciopercae, G. ouluensis*, *G. pungitii* and *G. salaris* [96]. *Gyrodactylus prostae* was positioned within the clade consisting of *Gyrodactylus* species of the *elegans*/*phoxini* group of the subgenus *G.* (*Gyrodactylus*) parasitising European cyprinids.

### 2 Dactylogyridae

Of the three dactylogyrid species found within the topmouth gudgeon native Asian range, i.e., *Ancyrocephalus parvae*, *D. squameus* and *B. obscurus*, two have been recorded in Europe. *Ancyrocephalus parvae* was observed solely in fish from Niushan Lake in China (native range), infecting 80% of fish with a mean abundance of 9.8 and an intensity of infection ranging from 1 to 27 (Table 1).

*Dactylogyrus squameus* was found rarely on the Dnieper River (Ukraine), Kyjovka River and the Melanbon borrow pit (Czech Republic), with a maximum of two parasites per fish (Table 1). Similarly, only two specimens of *D. squameus* have been recorded on native fish at Niushan Lake in China (Table 1). Measurements of the hard parts of the haptor and copulatory organ of *D. squameus* were higher in fish from its non-native European range (Supplementary Table S2, Fig. 1). Our specimens (Accession Nos. OQ598693 and OQ598695 for 18S and 28S) displayed low intraspecific 28S rDNA variability (*p*-distance = 0.1%) when compared with native specimens retrieved from GenBank (KX812459, Yingjiang of Yunnan, China, 28S), but the same sequence variability was observed for 18S-ITS1 rDNA when compared with specimens obtained in the Czech Republic (AJ564156; [78]).

**Figure 6:**
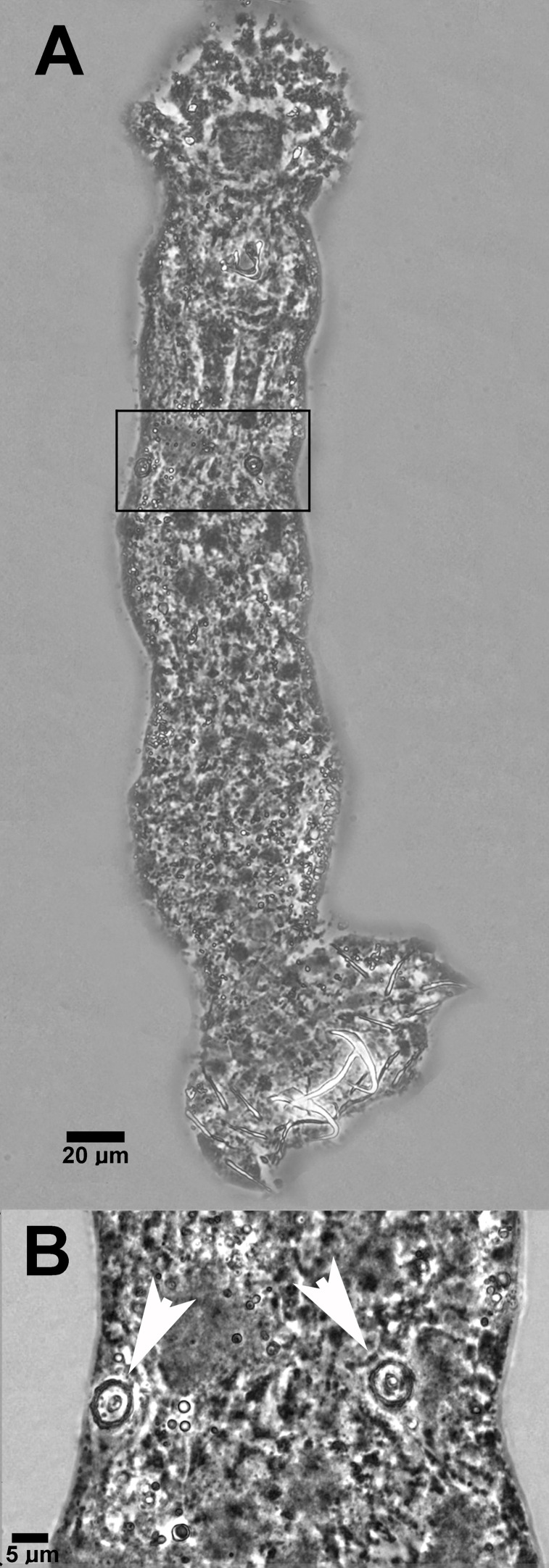
Photomicrograph of *Bivaginogyrus obscurus* ex *Pseudorasbora parva* (A) in its native Asian range (Niushan Lake, China), with the detail showing the two vaginae (B, white arrows).

In its non-native range, *B. obscurus* (Fig. 6) parasitised fish in the Dnieper River and the Melanbon borrow pit, with a prevalence of up to 40%, a mean abundance of 0.5 and a maximum intensity of infection of three parasites per host in the Melanbon borrow pit (Table 1). While no fish were infected at Bao’an Lake, all fish from Niushan Lake were infected at a mean abundance of 7.2 and an intensity of infection ranging from 1 to 14 parasites per host. Measurements for *B. obscurus* were higher in fish from its non-native European range (Fig. 1; Supplementary Table S2). A 789 bp nucleotide sequence of partial 28S rDNA, and a nucleotide sequence representing a 1006 bp rDNA fragment spanning partial 18S rDNA (489 bp) and the ITS1 (517 bp) sequence have been deposited in GenBank under accession numbers OQ598692 and OQ598694. No sequence variability was observed between specimens from the Dnieper River and the Melanbon borrow pit for the partial 28S or 18S–ITS1 rDNA sequences. A BLASTn search of both gene fragments revealed no identical hits with entries in GenBank (February 2023). The closest hit for the 28S rDNA sequence was for *D. primarius* (92.45%; OP320903; Chang *et al.*, unpublished) described from Chinese hooksnout carp *Opsariichthys bidens* in China, and for *D. gobiocypris* parasitising the gills of a rare minnow *Gobiocypris rarus* in China using the 18S–ITS1 sequence (89.94%; OP441417; [18]).

#### *Phylogenetic position of* B. obscurus *within Dactylogyridae*

**Figure 7:**
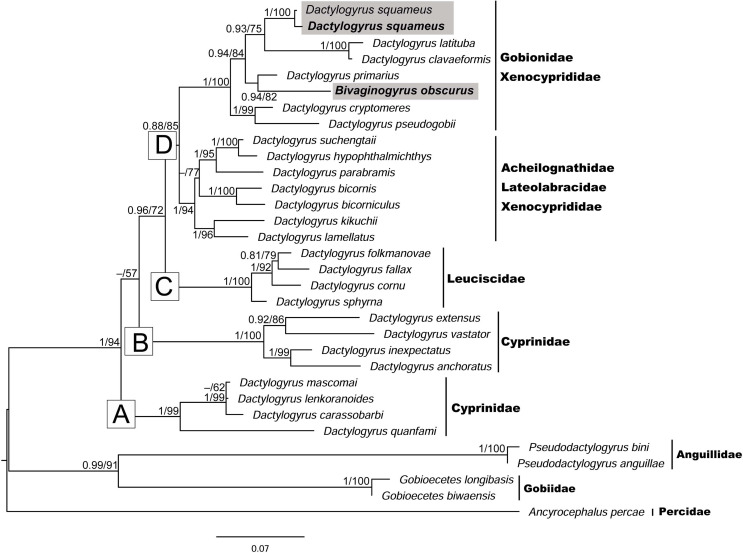
The phylogenetic relationships of *Bivaginogyrus obscurus* parasitising *Pseudorasbora parva* within Dactylogyridae. The phylogenetic tree is inferred from a Maximum Likelihood (ML) analysis of the 763 bp alignment of partial 28S rDNA sequences. Numbers along the branches represent Bayesian posterior probability/ML bootstrap support (only values > 0.70 for BI and > 50% for ML shown). Newly obtained sequences from the present study are in bold. Sequences of *Dactylogyrus* species parasitising *P. parva* are highlighted in grey. The branch length scale bar indicates the number of substitutions per site.

BI and ML analyses both provided trees with identical topologies (Fig. 7). The phylogenetic reconstruction divided all taxa into four strongly supported clades and revealed the clear inclusion of *B. obscurus* within *Dactylogyrus* species (Fig. 7). *Bivaginogyrus obscurus* appears to be most closely related to *Dactylogyrus* species belonging to clade D (lineage IV sensu Šimková *et al.* [81]), comprising Eurasian species parasitising Gobionidae, Xenocyprididae, Acheilognathidae and Lateolabracidae. This clade is divided into two well supported groups, whereas the group including *B. obscurus* comprises three species (*D. latituba*, *D. claveaformis* and *D. primarius*) parasitising sharpbelly carp *Hemiculter leucisculus* and *O. bidens* (Xenocyprinidae) in China, and the three *Dactylogyrus* species (*D. squameus*, *D. cryptomeres* and *D. pseudogobii*) parasitising Eurasian Gobionidae (topmouth gudgeon, gudgeon *Gobio gobio* and Chinese false gudgeon *Abbottina rivularis)*.

## Discussion

In its native range, the topmouth gudgeon is parasitised by three dactylogyrid and three gyrodactylid monogeneans. Of these, two dactylogyrids, *D. squameus* and *B. obscurus*, and two gyrodactylids, *G. gobioninum* and the *G. pseudorasborae* n. sp. described in this study, were co-introduced into Europe. To date, *A. parvae* and *G. parvae* have only been reported from the hostʼs native Asian range, while the three gyrodactylid species, including *G. prostae* (this study), *G. cyprini* and *G. katharineri* [48], were acquired once in Europe. Rare occurrence of native monogeneans along with the relatively low prevalence and abundance support the Enemy Release Hypothesis [87]. This theory suggests that some species may be temporarily advantaged by release of parasites and pathogens that are numerous in the native population but far less so in the new non-native population, contributing to their invasion success [69, 87]. Accordingly, avoidance of high parasite infections in the non-native range may possibly represent one of the factors contributing to the topmouth gudgeonʼs invasion success in Europe.

### Distribution of co-introduced monogeneans

Occurrence of *D. squameus* and *B. obscurus* was confirmed in both regions of this study, i.e., the Lower Dnieper irrigation canal in Ukraine and the basins of the Lower Morava and Dyje Rivers (Middle Danube watershed) in the Czech Republic. Previous European records of *D. squameus* include lentic water bodies in the Dyje River floodplain [62] and along the Morava River [78] in the Czech Republic, Ticino River in Italy [23] and a fish pond near Kyiv (Dnieper river basin) in Ukraine [94]. All reports show accidental occurrence of the parasite species rather than common infection, which agrees both with our results, showing low prevalence and abundance in both regions (Danube and Dnieper basins), and with the infection of native topmouth gudgeon observed in Niushan Lake in China (Table 1). Likewise, a low intensity of infection (1–3) was observed at three native sites in Japan, despite a higher prevalence (25–100%), indicating that infection of topmouth gudgeon with *D. squameus* is naturally low, potentially limiting the probability of co-introduction into new areas. Though our results do not extend the distribution of the parasite to a wider scale (river basin), they confirm that *D. squameus* is maintained in the host population for a long time, even at low prevalence and intensities of infection.

Natural infection by *B. obscurus* is apparently higher than by *D. squameus*, with all fish in Niushan Lake in China being infected with a mean abundance of 7.2 (Table 1), and a similar prevalence and abundance found in Lake Kasumigaura in Japan [57]. Likewise, a 100% prevalence was recorded in congeneric Japanese moroco *Pseudorasbora pumila pumila* in Utabi and Ymabuse ponds in Japan [57]. Nevertheless, reports of *B. obscurus* introduction into Europe are scarce, with the species previously reported solely from ponds in the Dnieper River basin near Kyiv [94]. Thus, our data extends the European distribution of *B. obscurus* to the Dyje basin (Danube watershed) in the Czech Republic, a distance of over 1,000 km, though the frequency of occurrence and loading remain relatively low. This recent finding of the species may suggest a new introduction event of topmouth gudgeon, possibly as a pest transported along with commercially important fish species.

Unlike dactylogyrids, *Gyrodactylus* parasites native to topmouth gudgeon were found at all sampling sites, often at high prevalence, including a new species, *G. pseudorasborae* n. sp. (see Table 1), indicating its wider distribution in Europe. While frequent occurrence of Asian *G. gobioninum* was observed in a Bulgarian fish farm [48], no further European records of this species have been published since that time. However, the taxonomy of *G. gobioninum* is still unresolved and probably involves a group of species [32]. Absence of *G. pseudorasborae* n. sp., as well as any dactylogyrids, in topmouth gudgeon from the Bulgarian fish farm may indicate that those fish were introduced from a source different from that of fish inhabiting lentic water bodies along the Morava, Dyje and Dnieper Rivers.

### Description of a new parasite species in its host’s non-native range

One side-effect of species introductions is the co-introduction of their parasites, and these species may receive more attention in their area of introduction than their native area. Thus, it is not unusual that some hitherto undescribed species is found and described in the host’s non-native range. As an example, the monogenean *Dactylogyrus extensus* was first described from common carp *Cyprinus carpio* in North America, though later phylogenetic studies indicated that the parasite is very likely of Eurasian origin [78], while cryptic diversity of *G. nebulosus* led to the description of *G. melas* from American bullhead *Ameiurus melas* in its non-native European range [64]. Similarly, thanks to more advanced imaging techniques and genetic methods, *G. pseudorasborae* n. sp. was distinguished as a separate species, despite its high morphological similarity with *G. parvae*. High genetic similarity with Chinese *Gyrodactylus* sp. obtained from topmouth gudgeon (99.57%; Chang *et al.*, unpublished), morphological consistence between European and our own data from two Chinese populations (Figs. 2 and 3) and the phylogenetic positioning of *G. pseudorasborae* n. sp. as a sister species to *G. parvae*, a native parasite of topmouth gudgeon, all support our suggestion that *G. pseudorasborae* n. sp. is of Asian origin and was introduced into Europe along with its fish host.

### Parasite acquisition in host’s non-native range

Parasite acquisition occurs relatively often after the introduction of a new host species into a novel environment, with the local parasite species acquired by invaders usually being those with low host specificity or those infecting phylogenetically related species [68]. Monogenean parasites are often host specific, particularly those of the Dactylogyridae [51, 79]; thus, it is no surprise that no local dactylogyrid species have been found to infect topmouth gudgeon in Europe. Gyrodactylidae are generally less specific and include species from strict specialists to generalists infecting fish over different families and orders [4]. Based on the published data, *G. prostae* appears able to infect a range of cyprinid species [29, 54], and infection of another cyprinid such as topmouth gudgeon would therefore be expected. It is rather surprising, therefore, that records of topmouth gudgeon infection by less specific local gyrodactylids are so rare (see Margaritov and Kiristis [48] for an exception) and that fish from only one locality in our study were infected with local gyrodactylid species, at relatively low prevalence and abundance. These results correspond to the findings of Margaritov and Kiristis [48], who reported only accidental occurrence of *G. cyprini* and low parasite loading by *G. katharineri* acquired on a Bulgarian fish farm, in contrast to the higher infection load in co-introduced *G. gobioninum*.

### Do parasites grow better in their non-native range?

Better performance of invasive species compared to their conspecifics in native ranges has been documented for a wide range of organisms, though this pattern is not universal and many other species largely perform the same across ranges. This increase in the size of invasive species has been promoted as one of the factors contributing to their invasion success [67]. Although neither of the parasites found in this study are considered invasive species, the length of the attachment apparatus hard parts and, in the case of dactylogyrids, of the copulatory organ, was generally larger in the introduced parasites. Previous studies have attributed the size of the haptoral hard parts in monogeneans to the size of the host [7, 51] or water temperature [21, 53]. In temperate zones, many gyrodactylids are cold-water preferring parasites and, accordingly, their abundance and the size of the haptoral hard parts does indeed tend to decrease with water temperature [21, 53]. This association may explain the significantly larger worms found in the coldest month sampled in this study (April, water temperature 14 °C) compared to other samples (Fig. 2) and the similar values between Chinese (native) and Ukrainian (non-native) parasites, both being collected in June. Nevertheless, larger mean measurements were observed across all samples from the non-native range, irrespective of season (Table S1). In contrast to gyrodactylids, studies have shown that the size of hard parts in dactylogyrids tends to reflect their host size [51]. On the other hand, host size could not explain the larger size of other dactylogyrids co-introduced to Europe from North America, i.e., *Onchocleidus dispar* and *Actinocleidus recurvatus* [63], as the host displayed a much slower growth rate and smaller adult size than fish in native range [19]. In comparison, the topmouth gudgeon collected in Europe were much larger than those from its native China (Table 1), suggesting that the species may perform better in its non-native range as space competition for gill parasites is lowered (absence of *A. pseudorasborae*), thus resulting in larger individuals. It is also possible that parasites co-introduced to Europe were those with larger attachment apparatuses, and that these better survive transportation and go on to establish populations in the host’s new range.

### *Phylogenetic positioning of* Bivaginogyrus obscurus *within Dactylogyridae*

*Bivaginogyrus obscurus* was originally described as a *Dactylogyrus* species from topmouth gudgeon in the Amur River basin [27]. Owing to its unique feature of possessing two vaginae (Fig. 6), however, Gusev and Gerasev [28] established a monotypic genus, i.e., *Bivaginogyrus*, for the species. Recent investigation of endemic gobionid fish in Japan, however, led to the description of three new *Bivaginogyrus* species infecting two *Gnathopodon* species [61]. Similarly to their observation, our phylogenetic analysis indicated inclusion of *B. obscurus* within *Dactylogyrus* species, with *B. obscurus* clustered with *Dactylogyrus* species parasitising Gobionidae and Xenocyprididae, including *D. squameus*, another dactylogyrid species infecting topmouth gudgeon (Fig. 7). Even though several studies have suggested a monophyletic origin of the *Dactylogyrus* (e.g., [78, 79]), more recent studies present data supporting its paraphyly by representatives of *Acolpenteron*, *Dogielius* and *Dactylogyroides* [1, 38]. This is consistent with our own results, with *Bivaginogyrus* being in a sister relationship with *D. primarius* infecting xenocyprid fish. As with *Acolpenteron ureteroecetes*, *B. obscurus* was included within clade G sensu Aguiar *et al.* [1] (clade IV sensu Šimková *et al.* [81]). Our results, therefore, further support the recent suggestions of a paraphyletic origin of the *Dactylogyrus*, suggesting the need for detailed phylogenetic studies including a wider range of dactylogyrid genera, as recommended by Kmentová *et al.* [38].

## Conclusions

Our results show that topmouth gudgeon, one of the most successful freshwater fish invaders in Europe, appears able to avoid high parasite infections in its non-native range. The fish were less parasitised by their native, co-introduced parasites compared to their native conspecifics, and acquisition of local parasite species occurred only rarely. Release from parasites has been considered one of the factors affecting successful establishment of non-native species and their subsequent invasiveness [87], which may well be applicable for topmouth gudgeon. A relatively low monogenean prevalence and abundance found in our study further corresponds to the generally low infection load by local species found over a wide range of European topmouth gudgeon populations [26].

## Supplementary material

The Supplementary materials of this article are available at https://www.parasite-journal.org/10.1051/parasite/2023024/olm. *Supplementary Table S1*: Comparison of morphometric parameters (mean, range; µm) for *Gyrodactylus pseudorasborae* n. sp. (Monogenea: Gyrodactylidae) collected from Pseudorasbora parva in various localities in Europe (non-native range) and China (native range). *Supplementary Table S2*: Comparison of morphometric parameters (mean, range; µm) for *Bivaginogyrus obscurus* and *Dactylogyrus squameus* (Monogenea: Dactylogyridae) collected from *Pseudorasbora parva* in various localities in Europe (non-native range), China (native range) and published data.
